# PANC-1 cancer stem-like cell death with silybin encapsulated in polymersomes and deregulation of stemness-related miRNAs and their potential targets 

**DOI:** 10.22038/ijbms.2021.54001.12136

**Published:** 2021-04

**Authors:** Fatemeh Khakinezhad Tehrani, Najmeh Ranji, Fatemeh Kouhkan, Simzar Hosseinzadeh

**Affiliations:** 1 Department of Biology, Faculty of Sciences, Rasht Branch, Islamic Azad University, Rasht, Iran; 2 Stem cell Technology Research Center, Tehran, Iran; 3 Department of Tissue Engineering and Regenerative Medicine, School of Advanced Technologies in Medicine, Shahid Beheshti University of Medical Sciences, Tehran, Iran

**Keywords:** Cancer stem cell, miRNA, Pancreatic cancer, Polymersome, Silybin

## Abstract

**Objective(s):**

Cancer stem cells (CSCs) have powerful self-renewal ability and tumor recurrence. Pancreatic ductal adenocarcinoma is a malignancy with high mortality rate and ˃5% survival. Silybin has anticancer and hepatoprotective properties. We loaded silybin in PEG400-OA (SPNs) and evaluated its cytotoxic effects on PANC-1 cells and PANC-1 CSCs.

**Materials and Methods:**

Spheroids from PANC-1 cells were obtained by the hanging drop method. Anti-proliferative and apoptotic functions of SPNs were evaluated in spheroids and non-spheroids with MTT, DNA fragmentation, PI and PI/AnnexinV assays. The expression of CD markers was assessed with flow cytometry. QRT-PCR was used to evaluate the expression of some miRNAs and targets.

**Results:**

IC_50_ of SPNs was identified to be 50 µg/ml, 45 µg/ml, and 42µg/ml, respectively after 24 hr, 48 hr, and 72 hr in PANC-1 treated cells. PI staining and PI/AnnexinV assay showed that ~20%, ~60%, and ~80%, of cells treated with 30, 50, and 60 µg/ml of SPNs were in sub-G1 and apoptosis phase, respectively. DNA degradation was confirmed after SPNS stimulation. CD24, CD44, and CD133 expression decreased after SPNs treatment both in PANC-1 spheroid cells and PANC-1 cancer cell line. Under-expression of onco-miRs (miR-21, miR-155, and miR-221), over-expression of several apoptotic potential targets of oncomiRs (Bax, Casp-9, and P53), over-expression of tumor suppressive-miRs (let-7b, miR-34a, and miR-126), and under-expression of Bcl-2 was found in SPNs-treated cells.

**Conclusion:**

We suggest that silybin encapsulated in polymersomes (SPNs) may be useful as a complementary agent for destroying both pancreatic cancer cells and pancreatic CSCs along with chemotherapeutic agents.

## Introduction

Pancreatic ductal adenocarcinoma (PDAC) is a malignancy with high lethality. PDACs are highly resistant to chemotherapy with a survival rate of less than 5% (1). Patients with pancreatic cancer have very limited options for treatment and this led to poor survival of pancreatic cancer (2). Cancer stem cells (CSCs) are a small population of cells with tumors that have the tumor-initiating ability as well as powerful self-renewal capacity. CSCs have the capability to renew themselves and express cell surface markers similar to normal stem cells. In addition, CSCs are resistant to radio/chemotherapy (3). Surface CD markers including CD44, CD24, CD133, and ESA/EpCAM are defined in pancreatic CSCs (4). It has been demonstrated that a subpopulation of CD133^+^ pancreatic cancer cells have CSCs characteristics and are highly chemo-resistance (2) Therefore, a suitable anticancer compound should be able to diminish CSCs as well as other cancer populations.

Silybin is the important component of *silymarin* (5) from *Silybum marianum* (milk thistle). The advantage of silybin and *silymarin* compared with other herbal compounds is that it has hepatoprotective effects, in addition to its anti-cancer, anti-inflammatory, and antioxidant activities (6). It is noteworthy that anticancer chemotherapy induces significant liver injury (7). Thus, it is suggested a hepatoprotective anticancer complementary can be useful along with anticancer chemical agents. However, silybin has one major problem: low solubility in body fluids (8), similar to other herbal compounds such as curcumin (9). 

Lipid-based drug nanocarriers are already approved for nanomedical aims and in clinical trials. These carriers are synthesized to load drug substances in their aqueous core. Polymersomes can be generated in different sizes, from tens of nm up to μm ( giant polymersomes) (10). Polymersomes are similar to liposomes, but they are more stable and storable nanocarriers compared with liposomes. Unlike micelles, polymersomes can be hydrophilic and hydrophobic substances (11). In our previous study, our team synthesized nanocarriers from PEG400 and oleic acid, which have physicochemical characteristics of this polymersome (12).

MicroRNAs (miRNAs or miRs) are a group of silencing RNAs (13) that suppress translation and induce mRNA degradation (14). Abnormal up/down-regulation of miRNAs has been revealed in different cancers (15,16). MiRNAs seem to play an essential role in self-renewal and differentiation of stem cells through negative regulation of “stem cell genes” expression (17). Recently, the role of miRNA in CSCs, as the origin of cancerous cells, has been found to be considerable (18). MiR-21, miR-221, miR-34, and let-7 family were found to play a role in regulating pancreatic CSC (19). MiR-34a induced programmed cell death in pancreatic cancer and colon cancer through p53. After tumor sphere-forming in CD44^+^/CD133^+^ pancreatic cells, high levels of Notch/Bcl-2 and loss of miR-34 have been identified (18). Liu *et al*. revealed that miR-34a, as a tumor suppressor miR, can inhibit prostate CSCs and directly under-express CD44 marker in these CSCs (19). LIN28B, an RNA-binding protein, represses let-7b expression and stimulates the proliferation and invasion of CD44^+^/LIN28B^+^ human pancreatic CSCs (1). Overexpression of Let7 led to esophageal cancer stem cell sensitivity to chemotherapy through Wnt signaling inhibition (20). Therapy with antagomiR-126 led to *in vivo* decrease of leukemia stem cell (LSCs), maybe via decrease of the quiescent cells (21). Decrease of miR-126 expression in acute myeloid leukemia (AML) cells declined cell growth via increase of apoptosis (22). The use of antagomir-221 in pancreatic CSCs has led to a significant reduction in cell number and differentiation (23). Knockdown of miR-21 decreased CD133^+^ subpopulation and spheroid formation in ovarian teratocarcinoma stem cells (24).  Overexpression of miR-21 and miR-155 was associated with poor prognosis in a large number of patients with pancreatic cancer (25).

Nowadays, in clinical practice and laboratory studies it has been demonstrated that a single treatment method might be not efficient to overcome heard diseases (such as cancers) due to their physiological (26) and genetic complexity. Therefore, combined or multiple therapies can be used as effective strategies to therapy (26). Silybin as a herbal component can have potential activity in combined therapies. For example, a study has shown that the bioavailability of paclitaxel was increased by silybin compared with that in the control group (27). Also, a study showed that curcumin as a herbal component could increase the antibacterial effect of ciprofloxacin on ciprofloxacin-resistant isolates of *P. aeruginosa* partly through underexpression of efflux pump genes including *mexX* and *oprM* in a synergic manner (9). However, combined therapies possess some drawbacks that might limit their routine clinical administration. For example, drug formulation properties such as drug release rate, adaptation rate to shape and anatomy of the desired site, biodegradability, and biocompatibility are important for combined therapy (26). In our study, we used polymersome nanoparticles as biodegradable and biocompatible nano-carriers to transfer silybin as a herbal compound with low solubility in water, into cells. Apoptotic and anti-proliferative effects of SPNs on stemness form of PANC-1 cells (spheroid) and non-spheroid form were evaluated. In addition, the expression level of some onco-/tumor suppressive- miRs (miR-34a, let-7b, miR-126a, miR-21, miR-221, and miR-155) and their potential targets were assessed in SPNs treated and untreated cells.

## Materials and Methods

***Preparation of silybin-encapsulated nanoparticles***

Silybin powder was obtained from Sigma-Aldrich Co. (Germany). Oloeyl Chloride and polyethylene glycol-400 (PEG_400_) were provided from Sigma-Aldrich Co. (USA). Tri-ethylamine was obtained from Millipore Co. (USA). The polyethylene glycol400-Oleate (PEG_400_-OA) was obtained through oleic acid (OA) and PEG_400 _esterification in the presence of chloroform and triethylamine at 25 °C for 4 hr. For purification of PEG_400_-OA, trimethylamine hydrochloride salt was filtered from the organic phase and chloroform was evaporated in a vacuum oven at 40 °C for 4 hr. Then, silybin was dissolved in acetone solution to obtain a weight/weight ratio of 1:6. After acetone evaporation, 1 mg/ml of Sil:PEG_400_-OA (1:6 ratio) in PBS was prepared to achieve the desired ratios. To confirm encapsulation of silybin into polymersomes, physicochemical measurements were performed (12). Then, silybin loaded in polymersomes was stored at 4 °C under dark conditions. 

***Cell culture***

PANC-1 pancreatic cancer cells (ATCC: CRL-1469) were obtained from the National Cell Bank of Iran (Pasteur Institute, Tehran, Iran). The cells were grown in DMEM containing 10% fetal bovine serum (FBS), 1% penicillin-streptomycin, and incubated at 37 °C in a humidified atmosphere containing 5% CO_2_. Chemical elements were obtained from GIBCO (USA).

***PANC-1 spheroids generation using hanging drop procedure***

Spheroid cells were generated from parental PANC-1 cells using the hanging drop method (26, 27). First, cancer cells were harvested using trypsin–ethylenediaminetetraacetic acid (EDTA) solution. 5x10^2^ cancer cells were suspended in 20 μl DMEM containing 10% FBS and antibiotics; then, the cells were spattered on the lid of a 10 cm petri dish (10 to 15 drops), the lid was then inverted. Hanging drop cultures were incubated under standard culture conditions (5% CO_2_, at 37 °C) for 2 days, the cell clusters were collected by pipetting the medium gently onto the lid of the dish. Each spheroid was gently caught and transferred to an untreated plate containing 10 ml DMEM and stored at 37 °C for 24, 48, and 72 hr. The inverted phase-contrast microscope (Olympus, Japan) was used for imaging spheroids. 

***Flow cytometry analysis of cancer stem cell (CSC) markers***

The SPNs-treated and untreated pancreatic cancer cells were cultured in 6-well microplates for 24 hr. After washing, these cells were incubated with Anti-CD133 monoclonal antibody (Thermo Fisher, USA) at 7 °C for 30 min. The washed cells were treated with a secondary human antibody (Thermo Fisher, USA). Then, the cells were incubated with Anti-CD24, Anti-CD44 for 30 min. Finally, the samples were analyzed by the FACSVerse™ instrument (Biosciences, USA). 

***SPNs cytotoxicity assay***

To determine the cytotoxic effects of SPNs on PANC-1 cells, MTT assay was performed. The cells were seeded into 96-well microplates at a density of 7x10^3^ cells/well and incubated at 37 °C overnight. Then, the cells were stimulated with different doses of SPNs (0–200 µg/ml) for 24, 48, and 72 hr at 37 °C containing 5% CO_2_. Subsequently, 10 µl MTT dye (5 mg/ml) was added to each well and incubated for 3 hr at 37 °C. After removing the supernatant, DMSO was added to dissolve the purple precipitate. To determine cell viability, the absorbance of treated and untreated cells was read at 570 nm using a microplate spectrophotometer (ELx800, BioTek, USA). Experiments were performed in triplicate and repeated at least three times.

***Cell cycle analysis by PI staining***

Cell cycle analysis was performed by flow cytometry based on PI staining protocol (28). PANC-1 cells were plated at a density of 0.5 × 10^6^ cells 6-well and cultured with 0, 30, 50, 60 µg/ml of SPNs for 24 hr. The cultured cells were collected, washed, and re-suspended in PBS. Ice-cold 70% ethanol was added to cells and then incubated at -20 °C for ≥ 2 hr. After cell washing, the fixed cells were stained with 0.5 ml cold Propidium Iodide (PI) (Sigma Aldrich, USA) solution with RNaseA (Sinaclon, Iran). After incubation at 37 °C for 30 min under dark conditions, the analysis of cell phases was performed using FACS calibur^TM^ instrument (Biosciences, USA) and FlowJo software V7.6.1 (Tree Star Co, USA). Experiments were repeated at least three times.

***Apoptosis analysis***

After 24 hr, SPNs-induced and uninduced PANC-1 cells (0, 40, 45, 47.5, 50, 60 µg/ml) were collected using trypsin. The cells were washed twice with ice-cold PBS (0.01 M, pH7.4). Binding buffer was added to the collected cells. The cells were incubated with Annexin V-FITC and PI dyes (Annexin V- fluorescein isothiocyanate (FITC) kit, Miltenyi Biotec, Germany) for 15 min at room temperature in the dark. A flow cytometer was used to analyze the percentage of early and late apoptotic cells. A total of 15,000 UN gated cells were analyzed by FACS calibur^TM^ instrument (Biosciences, USA) and FlowJo software V7.6.1. Experiments were repeated at least three times.

***DNA fragmentation assay***

After the abovementioned treatments, 1×10^6 ^SPNs-treated and untreated cells were collected. DNA was extracted from SPNs-treated and untreated cells using the Total Fragment DNA Purification Kit (Intron Biotechnology, South Korea). DNA fragmentation was analyzed with agarose gel electrophoresis (29). The samples (10 µg DNA) were subjected to electrophoresis on 2% agarose gels at 85V for 90 min. 

***The expression pattern of miRNAs in SPNs-treated cells***

The expression level of miR-126, miR-155, miR-21, miR-221, miR-34a, and let-7b in SPNs-treated and untreated PANC-1 cells were measured using quantitative RT-PCR. 1×10^6^ PANC-1 cells were briefly treated with 0 and 50 µg/ml of SPNs in a T-25 flask for 24 hr. Total RNA was isolated from the cells using RNX-PLUS (Sinaclon, Tehran, Iran). cDNA synthesis was performed by BONmiR™ qRT-PCR miRNA Detection Kit (Stem Cell Technology Research Center, Iran). SYBR Premix Ex Taq™ II kit (Takara, Japan) was applied for quantitative RT-PCR in the ABI^®^ StepOne^TM^ instrument. The amplification program was as follows: 95 °C for 10 sec, followed by 40 cycles at 95 °C for 5 sec, 62 °C for 20 sec, and finally, 72 °C for 30 sec. SNORD47 was used as an endogenous internal control for normalization of miRNA’s expression. The primer pairs (Stem Cell Technology Research Center, Iran) were listed in. The 2^−ΔΔCT^ equation was applied for analysis of the expression pattern of miRNAs. All reactions were run in triplicate at least three times.

***In silico analysis of miRNA’s potential targets***

*In silico* analysis was applied to determine the putative target genes of miR-126, miR-155, miR-21, miR-221, miR-222, miR-34a, and let-7b. Their targets were in proliferative and apoptotic pathways by algorithms such as miRWalk (http://zmf.umm.uni-heidelberg.de/apps/zmf/mirwalk2/), TargetScan (http://www.targetscan.org/vert_72/) and DIANA-microT (http://diana.imis.athena-innovation.gr/DianaTools/index.php?r=microtv4/index). 

***The expression pattern of miRNA’s potential targets in SPNs-treated cells***

Some potential targets of the abovementioned miRNAs were evaluated by quantitative RT-PCR. BONmiR detection kit was used for cDNA synthesis. SYBR Premix Ex Taq™ II kit (Takara, Japan) was applied for evaluation of the expression of some potential targets in the ABI^®^ StepOne^TM^ instrument. The amplification program was as follows: 95 °C for 15 sec, followed by 40 cycles at 95 °C for 5 sec and 60 °C for 30 sec. The primer pairs were listed in Table 1. B2M (beta2-microglobulin) gene as an internal control was used to normalize the expression of target genes. The 2^−ΔΔCT^ equation was applied to calculate the fold change of target genes in SPNs-treated cells compared with control. 

***Statistical analysis***

All experiments were repeated in at least three separate experiments, and results were measured as mean±standard deviations (SD). Unpaired t-test and ANOVA were used for statical analysis. Tukey’s *post hoc* analysis compares means of all compared. *A P*-value of 0.05 or less was considered to determine statistical significance.

## Results

***The effect of silybin loaded in polymersome nanoparticles (SPNs) on the viability*** ***of PANC-1 spheroid and non-spheroid cells***

Our experiments showed that silybin was loaded in polymersome nanocarriers (12). The optimal ratio of silybin to polymersomes was obtained to be 1:6 based on encapsulation efficiency and drug loading capacity. DLS results showed that SPNs had a PDI of 0.32 and a mean diameter of 219.2 nm. TEM image showed an average diameter of 221.7±59.23 nm. In addition, 1 mg/ml SPNs exhibited a drug loading of 15.81±0.57 and an encapsulation efficiency 94.86±0.07 (12).

**Figure 1:**
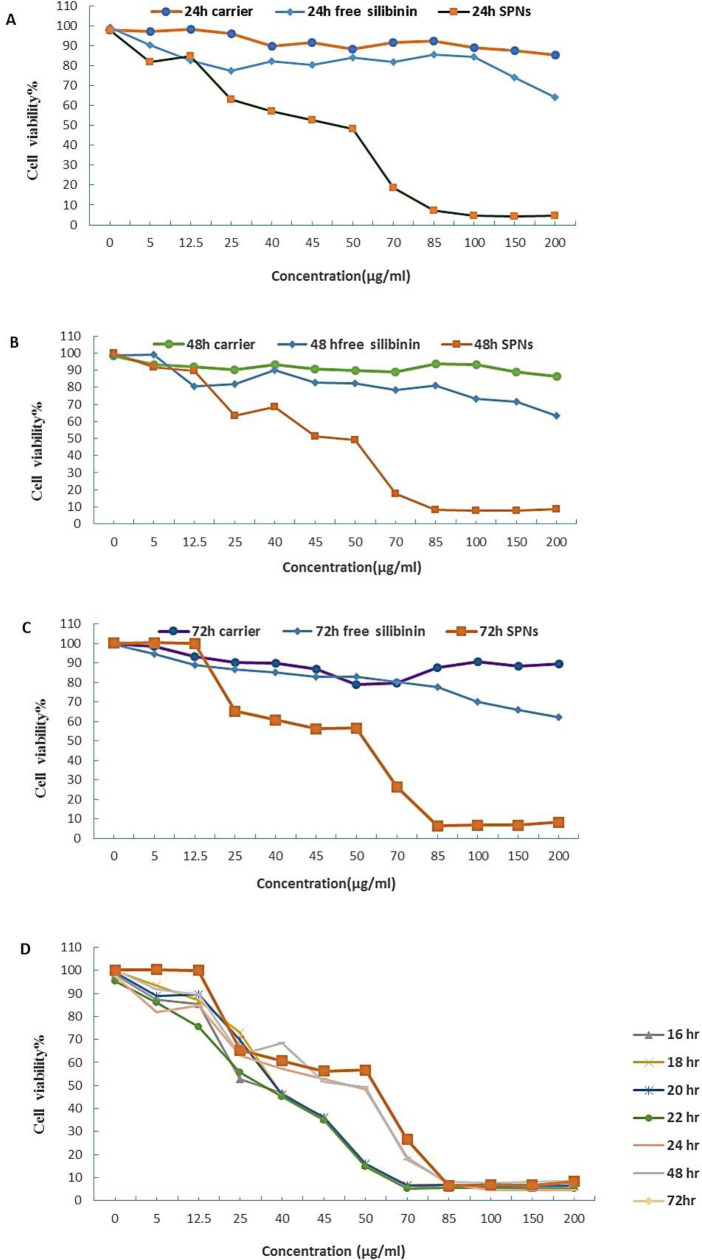
The effects of SPNs on cell viability of PANC-1 cells

MTT assay analysis revealed the dose-dependent and time-dependent effect of SPNs on PANC-1 cells for 24, 48, and 72 hr (Figures 1A-C). The half-maximal inhibitory concentration (IC_50_) value of SPNs was 50 µg/ml for 24 hr in PANC-1 cells. The IC_50_ value of SPNs on PANC-1 cells was 45 µg/ml after 48 hr and 42 µg/ml after 72 hr. Our results showed that viability of PANC-1 cells treated with 40 µg/ml and 60 µg/ml of SPNs was reduced by <60% and <20%, respectively after 16 to 72 hr (Figure 1D). In addition, MTT assay revealed that free silybin had lower cytotoxic effect on PANC-1cells compared with silybin loaded in polymersomes (SPNs). Furthermore, no significant cytotoxicity was observed for empty polymersome nanoparticles (up to 200 µg/ml) in 24 hr, 48 hr, and 72 hr treatments. 

***Presence of CD markers in SPNs-treated spheroid and non-spheroid cells***

**Figure 2:**
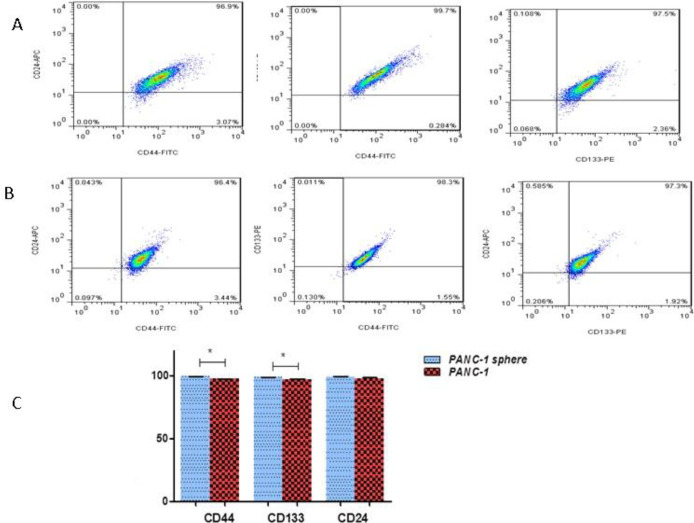
The expression levels of stem cell surface markers in PANC-1 spheroids and non-spheroids (the parental cells). Flowcytometric analysis of CD133, CD24, and CD44 in A) PANC-1 non-spheroids and B) PANC-1 spheroids, C) the percentages of CD133-, CD24- and CD44-positive cells in PANC-1 spheroids relative to the PANC-1 non-spheroids (parental cells without hanging drop)

In pancreatic cancer cells, three CD markers CD133^+ ^, CD24^+^, and CD44^+^ are known as stem cell surface markers. Flow cytometry analysis showed that percentages of CD133^+^, CD24^+^, and CD44^+^ were 97.2%, 97.7%, and 97.4% in PANC-1 paternal cells (without hanging drop procedure), respectively. Moreover, CD133^+^, CD24^+^, and CD44^+^ markers were expressed in 98.8%, 99.1%, and 99.4% of PANC-1 spheroid cells (after hanging drop procedure). Typical samples are shown in Figure 2.

**Figure 3:**
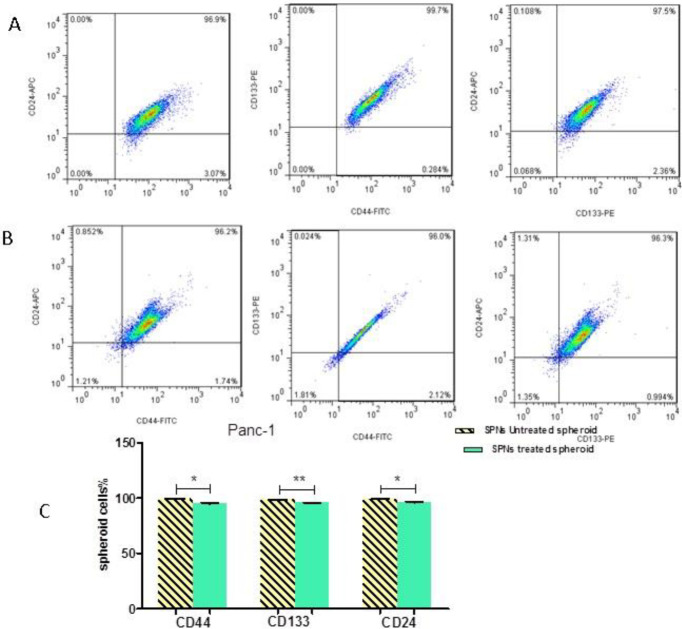
The expression levels of CD markers in SPNs-treated spheroids

**Figure 4:**
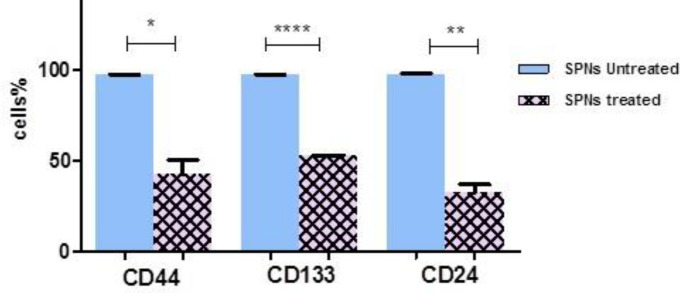
The expression levels CD markers in PANC-1 parental cells

Flow cytometry analysis revealed that 50 µg/ml of SPNs in spheroid cells decreased the percentage of stem cell surface markers (CD133, CD24, and CD44) compared with control (untreated cells) after 24 hr (Figure 3). However, SPNs (50 µg/ml) considerably decreased the percentage of these CDs in non-spheroid cells (without hanging drop) compared with control (non-spheroid cells not treated with SPNs) (Figure 4). 

***Effect of SPNs on the percentage of cell phases in PANC-1 cells***

**Figure 5:**
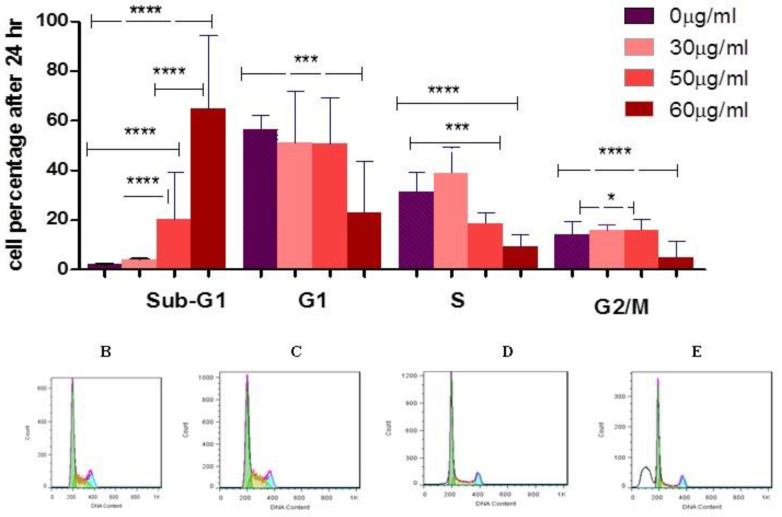
The percentage of cell phases in SPNs-treated and untreated cells

Our analysis showed S-phase arrest in SPNs-treated cells. SPNs significantly increased the number of arrested cells at the S-phase after 24 hr. Our findings showed that doses 30, 50, and 60 µg/ml of SPNs stimulated apoptosis (sub-G1) by 19.61%, 62.56%, and 85.01%, respectively (Figure 5).

***Apoptosis induction by SPNs in PANC-1 cells***

**Figure 6:**
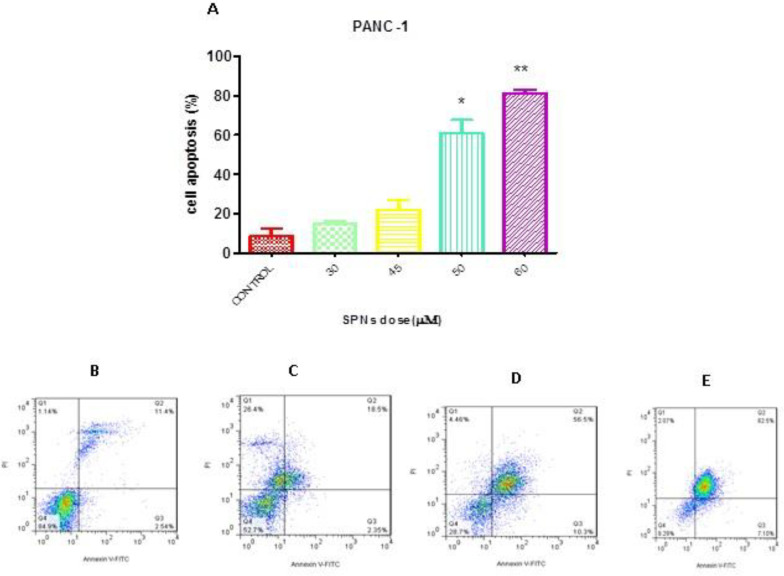
Flow cytometry analysis of SPNs-treated and untreated PANC-1 cells after Annexin V/PI staining

PI/Annexin assay was used to determine the percentage of apoptotic cells after treatment with different concentrations of SPNs (30 µg/ml, 50 µg/ml, and 60 µg/ml). Our results showed that AnnexinV^+^/PI^¯^ cells (early apoptotic cells, Q3) and AnnexinV^+^/PI^+^ cells (late apoptotic cells, Q2) increased after SPNs induction in a dose-dependent manner (Figure 6). Apoptosis was induced by ~60% in PANC-1 cells treated with 50 µg/ml SPNs. Moreover, 30 μg/ml and 60 µg/ml SPNs stimulated apoptosis in treated cells by <20% and >80%, respectively (Figure 6).

***Effect of SPNs on DNA fragmentation of PANC-1***

**Figure 7:**
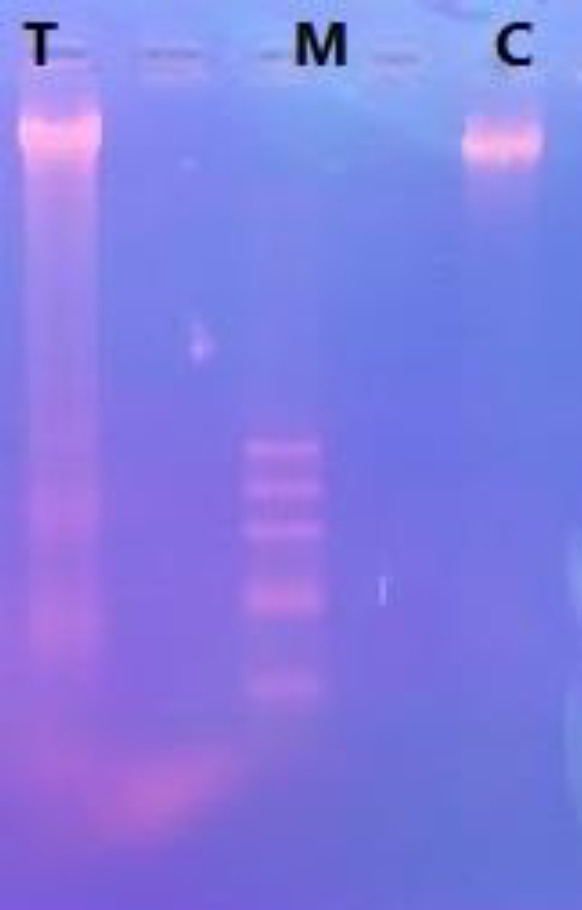
DNA fragmentation assay

Fragmented DNA, as a sign of apoptosis induction, was evaluated in SPNs-treated and untreated cells by electrophoresis of agarose gel. In PANC-1 cells, 50 µg/ml SPNs led to a DNA smear, while control cells had a band of DNA (Figure 7).

***Effect of SPNs on miRNA expression in PANC-1 cells***

**Figure 8:**
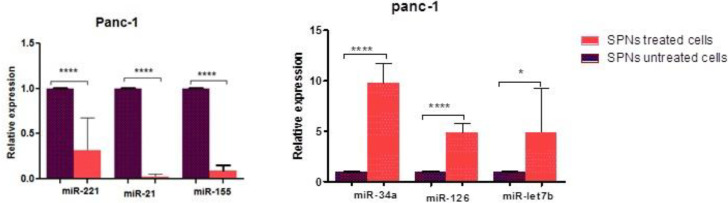
The mean value of miRNAs expression in SPNs-treated (50 μg/ml) and untreated PANC-1 cells

Our quantitative analysis revealed that miR-126, miR-34a, and miR-let7b in cells treated with 50 µg/ml SPNs were up-regulated by 4.7 to 9.7 folds compared with untreated cells. Moreover, in cells treated with 50 µg/ml SPNs, miR-155 and miR-21 were down-regulated by 0.02 to 0.3 folds compared with untreated cells (Figure 8).

***Expression of some potential targets of miRNAs***

**Figure 9:**
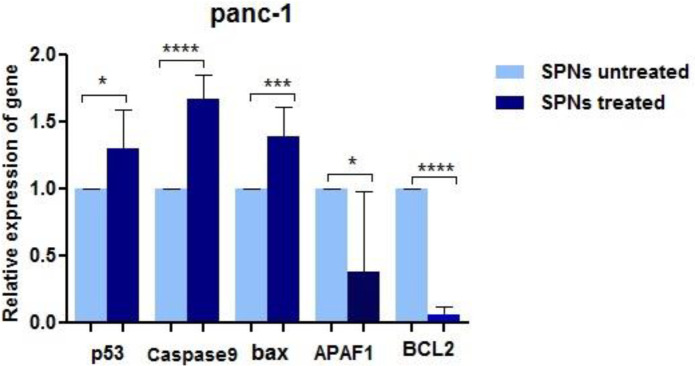
The expression levels of putative target genes of miRNAs. Under-expression of four apoptotic genes and overexpression of Bcl-2 in PANC-1 cells after stimulation of SPNs (50 μg/ml). The data are shown as mean±SD. Symbols denote significant differences between two cell groups (*P<0.05, ***P<0.001, and ****P<0.0001)

*In* *silico* prediction determined potential targets of the five abovementioned miRNAs in apoptotic and proliferative pathways (Table 2). Quantitative analysis by Q-RT-PCR revealed that four apoptotic genes *APAF1*, *p53, Bax*, and *CASP*-9 were overexpressed in PANC-1 treated cells with 50 μg/ml of SPNs compared with untreated ones. The anti-apoptotic gene BCL2 was significantly under-expressed in PANC-1 cells after SPNs induction (Figure 9).

**Table1:** Primers used for detection of mRNAs and miRNAs with Q-RT-PCR metohd

**Name**	**Sequence**
**hsa-mir-21-5p F**	**5'-GGCTTGTCAGACTGATGTTG** **-3'**
**hsa-miR-221-3p-F**	**5’-ATTCAGGGCTACATTGTCTG** **-3'**
**hsa-miR-222-3p –F**	**5’-ACGATGCCAGTTGAAGAAC** **-3'**
**hsa-miR-155-5p**	**5’-ACTTGGCTAATCGTGATAGG** **-3'**
**hsa-let-7b-F**	**5’-GCGTGAGGTAGTAGGTTGTG** **-3'**
**hsa-miR-34a-F**	**5’-ATGGTGGCAGTGTCTTAGC** **-3'**
**hsa-miR-126-3PF**	**5’-CAGCGTACCGTGAGTAATG** **-3'**
**SNORD47-F**	***5′-ATCACTGTAAAACCGTTCCA-3´***
**SNORD47-R**	***5′-GAGCAGGGTCCGAGGT-3´***
***Beta2M - F***	**5'-** ***ATGCCTGCCGTGTGAAC*** **-3'**
***Beta2M - R***	**5'-** ***ATCTTCAAACCTCCATGATG*** **-3'**
***P53 - F***	**5'-** ***GGAGTATTTGGATGACAGAAAC*** **-3'**
***P53 - R***	**5'-** ***GATTACCACTGGAGTCTTC*** **-3'**
***BCL2-F***	**5'-** ***GATAACGGAGGCTGGGATG*** **-3'**
***BCL2-R***	**5'-** ***CAGGAGAAATCAAACAGAGGC*** **-3'**
***EGF- F***	**5'-** ***TTTTGTTGTTCCTGCAGCCC*** **-3'**
***EGF- R***	**5'-** ***GCAAAATCATCAGCATGGACC*** **-3'**
***BAX-F***	***5'-CAAACTGGTGCTCAAGGC-3'***
***BAX-R***	***5'-CACAAAGATGGTCACGGTC-3'***
***APAF1-F***	***5'-GTCACCATACATGGAATGGCA -3'***
***APAF1-R***	***5'-CTATCCAACCGTGTGCAAA -3'***

Evaluation of cell viability by MTT assay. PANC-1 cells stimulated with polymersome nanoparticles (PEG400-OA), free silybin, and different doses (0–200 µg/ml) of silybin encapsulated in polymersomes (SPNs) for A) 24 hr, B) 48 hr, C) 72 hr, and D) cells stimulated with different concentrations of SPNs (0- 200 µg/ml) for 16-72 hr. The results are shown as the mean±SD

The percentage of stem cell CD markers in SPNs-treated spheroids PANC-1 spheroid cells; A) SPNs-untreated (50 µg/ml) spheroids and B) treated spheroids for 24 hr. C) The percentage of CD133, CD24, and CD44 markers in SPNs-treated spheroids and untreated spheroids. *P<0.05 and **P<0.01

A) The percentage of cells in different phases of the cell cycle in SPNs-treated and untreated PANC-1 cells. The percentage of sub-G1 (apoptotic cells), G1, S, and G2 phases in B) control cells, and in the treated cells with C) 30 µg/ml, D) 50 µg/ml, and E) 60 µg/ml SPNs. The results are shown as mean±SD. Symbols denote significant difference between different cell groups (*P<0.05, **P<0.01, ***P<0.001, and ****P<0.0001). SPNs: Silybin encapsulated in polymersome nanoparticles

A) Annexin V/PI staining demonstrated apoptosis percentage in SPNs-treated and untreated PANC-1 cells (30, 45, 50, and 60 μg/ml). Early apoptosis (Annexin+/PI-) and late apoptosis (Annexin+/PI+) enhanced in B) untreated cells, and treated with C) 45 μg/ml, D) 50 μg/ml, and E) 60 μg/ml for 24 hr. *P<0.05, **P<0.01

To evaluate DNA fragmentation, PANC-1 cells were stimulated with 50 µg/ml SPNs (T: test) relative to control cells (C: control) for 24 hr. DNA fragmentation assay was performed in agarose gel. M: Molecular weight marker (100 bp)

MiRNAs expression levels were evaluated in SPNs-treated and untreated cells and normalized to SNORD47 as an internal control. The data are shown as mean±SD. Symbols denote a significant difference between the two cell groups (*P<0.05, ****P<0.0001)

**Table 2:** Some potential targets of miRNAs in apoptotic and cell proliferation pathways

***microRNA***	***Potential target***	***Gene name***
***miR-21***	***APAF1***	***Apoptotic peptidase activating factor 1***
	***Tp53***	***Tumor protein p53***
	***CASP8***	***Caspase 8***
	***CASP9***	***Caspase 9***
	***CASP2***	***Caspase 2***
	***CASP3***	***Caspase 3***
	***CASP7***	***Caspase 7***
	***CASP10***	***Caspase 10***
	***DEDD***	***Death Effector Domain Containing***
	***FADD***	***Fas Associated via Death Domain***
	***PTEN***	***Phosphatase and Tensin Homolog***
	***IL-6***	***Interleukin 6***
	***IL-6R***	***Interleukin 6 Receptor***
	***TLR4***	Toll-Like Receptor 4
***miR-221***	***APAF1***	***Apoptotic peptidase activating factor 1***
	***BAG1***	***BCL2-associated athanogene 1***
	***BAD***	BCL2 associated agonist of cell death
	***BID***	BH3 interacting domain death agonist
	***CASP8***	***Caspase 8***
	***CASP9***	***Caspase 9***
	***CASP2***	***Caspase 2***
	***CASP7***	***Caspase 7***
	***CASP10***	***Caspase 10***
	***DAP***	***Death-Associated Protein ***
	***TRADD***	***TNFRSF1A Associated via Death Domain***
	***PTEN***	***Phosphatase And Tensin Homolog***
***.***	***FASLG***	***Fas Ligand***
***miR-155***	***APAF1***	***Apoptotic Peptidase Activating Factor 1***
	***P53***	***Tumor Protein P53***
	***BID***	BH3 Interacting Domain Death Agonist
	***BAG4***	***BCL2-Associated Athanogene 4***
	***DEDD***	***Death Effector Domain Containing***
	***TRADD***	***TNFRSF1A Associated Via Death Domain***
	***FADD***	***Fas Associated Via Death Domain***
	***FASLG***	***Fas Ligand***
	***CASP8***	***Caspase 8***
	***CASP9***	***Caspase 9***
	***CASP2***	***Caspase 2***
	***CASP6***	***Caspase 6***
	***CASP7***	***Caspase 7***
	***CASP10***	***Caspase 10***
	***PTEN***	***Phosphatase And Tensin Homolog***
	***TP63***	***Tumor Protein P63***
	***IL-6***	***Interleukin 6***
	***IL-6R***	***Interleukin 6 Receptor***
	***TLR4***	Toll-Like Receptor 4
***miR-126***	***AKT1***	Akt Serine/Threonine Kinase 1
	***BCL2L1***	Bcl2 Like 1
	***BCL2L12***	Bcl2 Like 1 ***2***
	***BCL2L14***	Bcl2 Like 1 ***4***
	***NOTCH1***	Notch Receptor 1
	***CD24***	Cd24 Molecule
	***CD34***	Cd34 Molecule
	***CDK4***	Cyclin-Dependent Kinase 4
	***CDK6***	Cyclin-Dependent Kinase 6
	***EGF***	Epidermal Growth Factor
	***EGFR***	Epidermal Growth Factor *** Receptor***
	***E2F3***	E2f Transcription Factor 3
***miR-34a***	***AKT1***	Akt Serine/Threonine Kinase 1
	***AKT2***	Akt Serine/Threonine Kinase 2
	***AKT3***	Akt Serine/Threonine Kinase 3
	***BCL2L11***	Bcl2 Like 11
	***SIRT1***	Sirtuin 1
	***NOTCH1***	Notch Receptor 1
	***CD24***	Cd24 Molecule
	***CD44***	Cd44 Molecule
	***E2F3***	E2f Transcription Factor 3
	***CDK4***	Cyclin-Dependent Kinase 4
	***CDK6***	Cyclin-Dependent Kinase 6
	***EGF***	Epidermal Growth Factor
	***EGFR***	Epidermal Growth Factor *** Receptor***
***Let7b***	***AKT1***	Akt Serine/Threonine Kinase 1
	***CDK4***	Cyclin-Dependent Kinase 4
	***CDK6***	Cyclin-Dependent Kinase 6
	***E2F2***	***E2f Transcription Factor 2***
	***MAP3K1***	***Mitogen-Activated Protein Kinase Kinase Kinase 1, E3 Ubiquitin Protein Ligase ***
	***CD34***	Cd34 Molecule
	***CD44***	Cd44 Molecule
	***SIRT1***	Sirtuin 1
	***EGF***	***Epidermal Growth Factor***
	***EGFR***	***Epidermal Growth Factor Receptor***
	***BCL2***	BCL2 Apoptosis Regulator
	***BCL2L10***	BCL2 Like 10
	***BCL2L12***	***BCL2 Like 12***
	***BCL2L13***	***BCL2 Like 13***
	***BCL2L14***	***BCL2 Like 14***

## Discussion

Silybin, as a herbal dietary supplement (28), is an effective chemo-preventive agent in various cancer types with inhibition of cancer cell growth (29), effects of which on CSCs need further evaluations. In our study, we showed that silybin encapsulated in SPNs diminished proliferation and increased apoptosis in PANC-1 cancer stem-like cells. Our analysis revealed that several stem cell CD markers, oncomiRs, and oncogenes were down-regulated and several tumor-suppressive miRs and tumor-suppressor genes were up-regulated after SPNs treatment.

CD44 is a CD marker with functions such as cell adhesion, cell growth, epithelial-mesenchymal transition (EMT), and tumor progression (30). CD44^+^ CD24^+^ epithelialspecific antigen pancreatic cancer cells demonstrated stem cell properties such as self-renewal, tumorigenic capacity, maintenance of tumor growth, and resistance to chemo- or radiation therapy. CD44^+^ CD24^+^ CD133^+^ cells exhibited biological properties of cancer stem-like cells (31). Previous studies found that chemo/radiation resistance in PDAC cells may be related to pancreatic CSCs. Our flow cytometry analysis revealed that the percentage of surface CD markers in SPNs-treated spheroid cells after 24 hr decreased slightly compared with untreated cells. After 72 hr, however, all spheroid cells treated with SPNs were destroyed. Therefore, SPNs may inhibit CD markers in these stem cells with increase in the duration of treatment. Therefore, as time passed, SPNs penetrated deep into spheroid cells and affected these cells. Moreover, SPNs reduced the aforementioned CD markers in PANC-1 un-spheroid cells greatly compared with untreated cells after 24 hr. Our results suggested that SPNs affect both PANC-1 cancer cells and PANC-1 cancer stem-like cells through decreasing CD surface markers CD24, CD44, and CD133.

Today, silybin is known as an anticancer agent. CSCs, as important sub-population of cancer cells, should be destroyed using effective anticancer agents. Silybin (150 µM) decreased HCT116 derived CD44^+^ cancer stem-like cells to ~48% after 48 hr (30). In another study, 500 and 1500 µmol/l silybin reduced stemness properties in 2D and 3D models of MDA-MB-468 after 48 hr, respectively (32). In our study, IC_50_ of SPNs on Panc-1 cancer cells was 50 µg/ml after 24 hr. Increase in treatment time with SPNs decreased IC_50_ of SPNs. Besides, the use of 40 µg/ml of SPNs on PANC-1 cancer stem-like cells destroyed spheroid cells within 48 to 72 hr. It seems that silybin encapsulated in polymersomes can be a promising strategy to overcome cancer stem cell growth in deep cancerous tissue in the future. Of course, this claim needs further experiments.

In a study on pancreatic cancer cell lines, silybin induced G1 arrest in AsPC-1 cancer cells, but not in PANC-1 and BxPC-3 cancer cells.  100 μM silybin induced apoptosis in about 13% of AsPC-1 cells, about 7% of BxPC-3 cells, and about 6% of Panc-1 cells after 24 hr (33). In a study, silybin (25-100μM) treatment for 24-72 hr caused cell growth inhibition of 27-77% in BxPC3 cells, and 22-45% in PANC-1 cells (34). Cell cycle analysis by PI staining and apoptosis evaluation by PI/Annexin V in our study showed similar results. SPNs could enter >60% cells to sub-G1 or induce apoptosis with a concentration of 50 µg/ml after 24 hr. At higher concentration (60 µg/ml) of SPNs, both experiments revealed a death percentage of >80% after 24 hr.

With tumor sphere-formation in CD133^+^/ CD44^+ ^MiaPaCa2 cells, loss of miR-34 and increase of Notch/Bcl-2 has been reported (17). Liu *et al*. revealed that miR-34a, as a tumor-suppressor miR, in prostate CSCs can inhibit cell growth and directly under-express the CD44 marker in these CSCs (35). miR-34a could reduce breast cancer stem cell properties and chemoresistance also, miR-34c was remarkably under-expressed in AML stem cells. In April 2013, miR-34 mimics in nanocarriers, as the first microRNA-associated therapeutic drug, was tested in a clinical trial (NCT01829971) (13). In our study, up-regulation of miR-34a, down-regulation of Bcl2, and decrease of CD markers in SPNs treated cells may represent an association between them. It seems that SPNs, through increase in miR-34a expression, suppress Bcl2 and cancer stem cell surface markers. LIN28B, an RNA-binding protein, represses the expression of *let-7b* and stimulates the proliferation and invasion of CD44^+^/LIN28B^+^ human pancreatic CSCs (1). Overexpression of *Let7* sensitized the esophageal CSCs to chemotherapies through Wnt pathway inhibition (20). The use of antagomiR-126 loaded in nanoparticles led to *in vivo* decrease of LSCs, probably by diminishing the quiescent stem cells (21). Attenuating the expression of miR-126 in AML cells decreased *in vitro* cell growth by apoptosis induction (22). Up-regulation of let7b and miR-126, as two tumor-suppressor and important miRNAs in inhibition of CSCs, was revealed in SPNs-treated PANC-1 cells. Let7 b and miR-126 may inhibit proliferation of SPNs-treated PANC-1 cells through under-expression of stem cell markers and anti-apoptotic genes as well as over-expression of apoptotic genes. 

The use of antagomir-221 in pancreatic cancer cells significantly reduced the fraction and differentiation of stem-like cancer cells (23). Knockdown of miR-21 decreased CD133^+^ population and sphere formation of ovarian teratocarcinoma stem cells (24). Overexpression of miR-155 and miR-21 is associated with poor prognosis in many patients with pancreatic cancer (25). In SPNs-treated PANC-1 cells, under-expression of miR-155, miR-21, and miR-222 in association with the decrease in CD markers CD24, CD44, and CD133 may indicate the importance of SPNs in inhibition of onco-miRs and stemness properties of PANC-1 cancer cells. 

In apoptosis induced by *Myc*, two apoptotic proteins Caspase-9 and Apaf-1 were identified to be the necessary downstream components of p53 (36). Bax, Bak, Casp-9, and Apaf1 are important compounds of the intrinsic pathway of apoptosis (37). It has been demonstrated that silybin stimulated under-expression of *Bcl*-2 and overexpression of *Bax* and *Casp*-8, *Casp*-9, and *BID* in MCF-7 breast cancer cells (5). Up-regulation of several members of the intrinsic pathway of apoptosis (*Casp*-9, *Bax*) and p53 as targets of under-expressed oncomiRs and down-regulation of *Bcl*-2 as the target of over-expressed tumor suppressive-miRs in PANC-1 cells treated with SPNs suggested the efficiency of silybin in cell growth inhibition via several mechanisms. 

## Conclusion

Our analysis revealed that silybin (50 µg/ml) encapsulated in polymersomes (SPNs) could induce death (~60%) in PANC-1 cancer cells. In addition, SPNs decreased stemness properties such as surface CD markers in PANC-1 spheroid and parental (without hanging drop procedure) cells. Our analysis showed that tumor-suppressive miRs, involved in suppression of CSCs, such as miR-34a and let-7b and miR-126 increased (~5 to ~10 folds) in SPNs (50 µg/ml)-treated cells, while effective onco-miRs in CSCs including miR-155, miR-21, and miR-221 were down-regulated (0.02 to 0.3 folds) in PANC-1 cells treated with SPNs (50 µg/ml) compared with untreated ones. Thus, silybin can be introduced as an anti-cancer stem cell agent. Lower dosage of silybin compared with previous studies may indicate the effectiveness of polymersomes in silybin transfer to cancer cells and depth of spheroids. Also, SPNs can suggest a promising strategy for the treatment of pancreatic cancers along with chemotherapeutic agents, since it has hepatoprotective activities in addition to anti-cancer and anti-cancer stem cell properties. 
